# N-truncated amyloid β (Aβ) 4-42 forms stable aggregates and induces acute and long-lasting behavioral deficits

**DOI:** 10.1007/s00401-013-1129-2

**Published:** 2013-05-18

**Authors:** Yvonne Bouter, Katharina Dietrich, Jessica L. Wittnam, Nasrollah Rezaei-Ghaleh, Thierry Pillot, Sophie Papot-Couturier, Thomas Lefebvre, Frederick Sprenger, Oliver Wirths, Markus Zweckstetter, Thomas A. Bayer

**Affiliations:** 1 Division of Molecular Psychiatry, Georg-August-University Goettingen, University Medicine Goettingen, von-Siebold-Strasse 5, 37075 Goettingen, Germany; 2 German Center for Neurodegenerative Diseases (DZNE), 37077 Göttingen, Germany; 3 Department for NMR-based Structural Biology, Max Planck Institute for Biophysical Chemistry, 37077 Goettingen, Germany; 4 SynAging, 54000 Nancy, France

**Keywords:** Pyroglutamate Abeta, Toxicity, Neuron loss, Degeneration, Transgenic mouse model, Spatial reference memory

## Abstract

*N*-truncated Aβ_4-42_ is highly abundant in Alzheimer disease (AD) brain and was the first Aβ peptide discovered in AD plaques. However, a possible role in AD aetiology has largely been neglected. In the present report, we demonstrate that Aβ_4-42_ rapidly forms aggregates possessing a high aggregation propensity in terms of monomer consumption and oligomer formation. Short-term treatment of primary cortical neurons indicated that Aβ_4-42_ is as toxic as pyroglutamate Aβ_3-42_ and Aβ_1-42_. In line with these findings, treatment of wildtype mice using intraventricular Aβ injection induced significant working memory deficits with Aβ_4-42_, pyroglutamate Aβ_3-42_ and Aβ_1-42_. Transgenic mice expressing Aβ_4-42_ (Tg4-42 transgenic line) developed a massive CA1 pyramidal neuron loss in the hippocampus. The hippocampus-specific expression of Aβ_4-42_ correlates well with age-dependent spatial reference memory deficits assessed by the Morris water maze test. Our findings indicate that *N*-truncated Aβ_4-42_ triggers acute and long-lasting behavioral deficits comparable to AD typical memory dysfunction.

## Introduction

Alzheimer disease (AD) is a progressive neurodegenerative disorder characterized by the presence of extracellular amyloid plaques composed of amyloid-β (Aβ) surrounded by dystrophic neurites and neurofibrillary tangles. The discovery that certain early-onset familial forms of AD may be caused by an enhanced production of Aβ peptides led to the hypothesis that amyloidogenic Aβ is intimately involved in the AD pathogenic process [58]. Besides Aβ peptides starting with an aspartate at position 1, a variety of different *N*-truncated Aβ peptides have been identified in AD brains. Ragged Aβ peptides, including a major species beginning with phenylalanine at position 4 of Aβ (Aβ_4-42_), have been reported as early as 1985 by Masters et al. [33]. Among different Aβ species present in AD plaques, Lewis et al. [31] demonstrated that Aβ_4-42_ is a relatively abundant species in AD, aged controls and vascular dementia patients. Using immunoprecipitation in combination with mass spectrometry, Portelius and colleagues [47] corroborated these earlier findings, reporting that Aβ_4-42_ is one of the major fractions in the hippocampus and cortex of AD patients. It has been demonstrated that *N*-terminal deletions, including Aβ_4-42_, enhance Aβ aggregation [45] and that the *N*-terminus specifies fibrillization behavior [17].

There is increasing evidence that the primary insult in AD is caused by oligomeric species derived from full-length Aβ_1-42_ impairing synaptic functions [14, 67]. In addition to soluble oligomers, β-sheet containing amyloid fibrils is also a highly toxic form of Aβ [16, 23, 59]. It has further been demonstrated that soluble oligomeric Aβ_1-42_, but not plaque-associated Aβ, correlates best with cognitive dysfunction in AD [3, 30]. Numerous variants of Aβ_1-42_ oligomers have been introduced and are currently being discussed as major factors in AD (reviewed in [3]). These include soluble Aβ_1-42_ dimers, trimers, tetramers and other variants, which have been demonstrated to be neuro- and/or synaptotoxic using cell or tissue culture models [29, 30, 43, 60, 61]. It has been argued that a variety of low and high molecular weight soluble Aβ_1-42_ aggregates, rather than just one particular type of oligomer, could trigger neuronal dysfunction [34]. However, a consensus is lacking as to which types of Aβ structures, dynamics and bioactivities are the causal link to AD [51]. In addition to the numerous variants of Aβ_1-42_ oligomers currently being discussed [3], there is substantial evidence that *N*-terminal truncated peptides play a key role in AD [20].

The aim of the present work was to elucidate the structure of Aβ_4-X_ aggregates and to study the potential acute and chronic effects of Aβ_4-42_ exposure in different model systems.

## Materials and methods

### Sample preparation

The amyloid β (Aβ) variants Aβ_4-38_, Aβ_4-40_, Aβ_4-42_, Aβ_pE3-42_ and Aβ_1-42_ were purchased from Peptide Specialty Laboratory (PSL, Heidelberg, Germany) and used without further purification. The peptide samples were prepared by first dissolving them in 1,1,1,3,3,3-hexafluoro-2-propanol (HFIP), flash-freezing in liquid nitrogen, and then lyophilizing them to completely remove the solvent. Lyophilized Aβ peptides were then dissolved in 100 mM NaOH at a concentration of 2 mg/mL, aliquoted in 50 μL volumes, flash-frozen in liquid nitrogen and stored at −80 °C until use.

### Thioflavin T (ThT) fluorescence measurement

Peptide samples of 40 μM concentration were prepared in HEPES buffer (25 mM, pH 7.4) containing 50 mM NaCl and 30 μM Thioflavin T (ThT). The kinetics of Aβ aggregation were then followed by real-time fluorescence emission measurement of ThT while the sample was kept at 37 °C and gently stirred. The excitation and emission wavelengths were 446 and 485 nm, respectively, with slits of 10 nm each.

### Transmission electron microscopy (TEM)

Peptide samples of 0.1 mg/mL concentration in HEPES buffer (25 mM, pH 7.4, 50 mM NaCl) were incubated at 37 °C with gentle stirring. After 3 days of incubation, samples were diluted, deposited onto carbon-coated copper mesh grids and negatively stained with 2 % (w/v) uranyl acetate. The excess stain was washed away, and the sample grids were allowed to air-dry. The samples were then viewed with a 120-kV transmission electron microscope.

### Circular dichroism (CD) spectroscopy

Samples of 0.2 mg/mL peptide concentration in phosphate buffer (20 mM, pH 7.2) were prepared. The far-UV CD measurements were conducted on a Chirascan CD spectrometer, with a 1-mm path length quartz cell, 1-nm bandwidth and 8-s collection time for each point at 0.5 nm steps between 190 and 260 nm. The buffer spectrum was also measured and subtracted from the spectra of Aβ peptides. The temperature-dependence of secondary structure was studied through far-UV measurements at three different temperatures, 20, 30 and 40 °C. The reversibility of temperature-induced changes was checked with an additional CD spectrum measured after cooling down from 40 to 20 °C. The CD spectrum of the cooled sample was taken after 5 min of equilibration at 20 °C. The 0.2 mg/mL Aβ samples in phosphate buffer (20 mM, pH 7.2) containing 25 mM NaCl were incubated at 37 °C for 3 days with gentle stirring. Following ThT fluorescence measurements which confirmed the presence of ThT-reactive aggregates in the peptide samples, the far-UV CD spectra were obtained at 20 °C as described above.

### Dynamic light scattering (DLS)

DLS experiments were performed at 20 °C on a DynaPro Titan (Wyatt Technology Corp., CA) instrument with a scattering angle of 90°. The samples were centrifuged at 16,000g for 15 min, and then the supernatant was taken for DLS measurements. The “monomeric” samples were prepared freshly in phosphate buffer (20 mM, pH 7.4) containing 25 mM NaCl at a peptide concentration of 0.2 mg/mL. The aggregated samples were prepared after incubation at 37 °C for 24 h, without further promotion of aggregation by agitation. The size distribution was determined by a constrained regularization method. The reported scattering intensities are from three separate measurements of the same sample.

### 1D ^1^H nuclear magnetic resonance (NMR) monomer consumption assay

1D ^1^H NMR spectra of the five different Aβ peptides were measured at 5 °C at 400 MHz ^1^H Larmor frequency. The samples contained 40-μM peptide in phosphate buffer (20 mM, pH 7.2) with 25-mM NaCl. The aggregation propensity of the Aβ variants were then studied through real-time 1D ^1^H NMR experiments at 37 °C continued for 14 h at 1-h intervals. The integrated intensity of ^1^H peaks at two regions (0.50–1.05 ppm, 6.50–8.00 ppm), after chemical shift using DSS (4,4-dimethyl-4-silapentane-1-sulfonic acid) as a reference standard, was then calculated. After normalization by the integrated intensity of the DSS peak at 0 ppm, the relative intensity of the peptide ^1^H peaks was used to probe peptide monomer consumption during the early phases of peptide aggregation.

### Western blot

Lyophilized Aβ peptides were dissolved in 10-mM NaOH at a concentration of 1 mg/mL, aliquoted in 50 μL volumes, flash-frozen in liquid nitrogen and stored at −80 °C until use. For Western blot analysis under reducing conditions, 1 μg peptides were loaded on 4–12 % VarioGels (Anamed), transferred to 0.45 μm nitrocellulose membranes and detected using the primary antiserum 24311 (pan-Aβ, 1:500) or monoclonal antibody 4G8 (Aβ17-24, Signet; 1:500). For Western blotting of mouse brain, whole brain SDS lysates were used. Running and transfer buffers were applied according to the manufacturer. The blots were developed using enhanced chemiluminescence according to the manufacturer (Roth). Horse radish conjugated swine anti-rabbit antibody was used as a secondary antibody (1:3,000, Dianova).

### Neuronal culture

Cortical neurons from embryonic day 16–17 Wistar rat fetuses were prepared as previously described [46]. In brief, dissociated cortical cells were plated at 50,000 cells/well in 48-well plates precoated with 1.5 mg/mL polyornithine (Sigma). Cells were cultured in a chemically defined Dulbecco’s Modified Eagle’s/F12 medium free of serum (Gibco) and supplemented with hormones, proteins and salts. Cultures were kept at 35 °C in a humidified 5 % CO_2_ atmosphere, and at 6–7 DIV, cortical population was determined to be at least 97 % neurons by immunostaining as done previously [71]. At 6 DIV, the medium was removed and cortical neurons were incubated for 24 h with vehicle (cell culture medium) or Aβ peptides (dissolved in cell culture medium) at the indicated concentrations.

### Cell viability measurement

Following a 24-h incubation of primary cortical neurons with Aβ peptides, cell viability was determined using a calcein-AM assay (Invitrogen, Molecular Probes). Briefly, cells were washed twice with PBS and incubated to protect from light for 30 min at room temperature in the presence of 2 μM calcein-AM solution prepared in PBS. Cells were then washed twice with PBS and incubated for 15 min at room temperature in PBS containing 1 % Triton X-100 (v/v). The level of calcein fluorescence was monitored by fluorescence emission at 530 nm after exciting at 485 nm, using a Fluostar microplate reader (BMG-Labtechnologies, France).

### Intracerebroventricular injection of soluble Aβ

Male C57BL/6 J mice (12-week old, Janvier, Le Genest-St-Isle, France; *n* = 6 per treatment group) were housed five to six per cage with free access to food and water, and were kept in a constant environment (22 ± 2 °C, 50 ± 5 % humidity, 12-h light cycle). Under anesthetization, freshly prepared Aβ peptides (50 pmol in 1 μL; 0.1 M phosphate-buffered saline (pH 7.4)) or vehicle (0.1 M phosphate-buffered saline) were injected into the right ventricle, with stereotaxic coordinates from the bregma (AP −0.22, L −1.0 and D 2.5 in mm). Intracerebroventricular (icv) injections were made using a 10-μl Hamilton microsyringe fitted with a 26-gauge needle. Four days following icv infusion of Aβ peptides, working memory was assessed using the Y-maze test.

### Working memory by the Y-maze task

Immediate spatial working memory performance in male C57BL/6 J wildtype mice (12-week old, Janvier, Le Genest-St-Isle, France; *n* = 6 per treatment group) was assessed by recording spontaneous alternation behavior in a Y-maze as described previously [55, 71]. The Y-maze task was carried out on day 4 after soluble Aβ application. The maze was made of opaque plexiglas and each arm was 40-cm long, 16-cm high, 9-cm wide and positioned at equal angles. Mice were placed at the end of one arm and allowed to move freely through the maze during a 5-min session. The series of arm entries were recorded visually and arm entry was considered to be completed when the hind paws of the mouse were completely placed in the arm. Alternation was defined as successive entries into the three arms on overlapping triplet sets. The percentage alternation was calculated as the ratio of actual (total alternations) to possible alternations (defined as the number of arm entries minus two), multiplied by 100.

### Generation of transgenic mice

The cDNA coding for Aβ_4-42_ was inserted into the Thy1 expression construct and verified by sequencing. The transgenic founder mice were generated by male pronuclear injection of fertilized C57BL/6 J oocytes. The resulting offspring were further characterized for transgene integration by PCR analysis, and after crossing to C57BL/6 J wildtype mice, for transgene expression by RT-PCR. Line 2, the line with highest transgene mRNA expression, was selected for further breeding (thereafter named Tg4-42). All animals were handled according to German guidelines for animal care. The mean age of the mice tested were 3 ± 1, 8 ± 1 and 12 ± 1 months.

### Immunohistochemistry and histology

Mice were killed via CO_2_ anesthetization followed by cervical dislocation. Brain samples were carefully dissected and post-fixed in 4 % phosphate-buffered formalin at 4 °C. Immunohistochemistry was performed on 4-μm paraffin sections. The following antibodies were used: 24311 (1:500; rabbit polyclonal against pan-Aβ), Aβ42 (1:500; Synaptic Systems, rabbit polyclonal specific for the C-terminus of Aβ42), synaptophysin (1:500; Synaptic Systems, monoclonal), GFAP (1:500; Chemicon), Iba1 (1:500; Waco). Biotinylated secondary anti-rabbit and anti-mouse antibodies (1:200) were purchased from DAKO. Staining was visualized using the ABC method, with a Vectastain kit (Vector Laboratories) and diaminobenzidine as chromogen. Counterstaining was carried out with hematoxylin. For DAPI staining sections were deparaffinized and washed in PBS followed by incubation in 4′,6-diamidine-2′-phenylindole (DAPI, 1 μg/ml) for 1 min. Embedding was performed in aqueous fluorescent mounting medium (DAKO).

### Quantification of neuron numbers using unbiased stereology

Mice were anaesthetized and transcardially perfused with 4 % paraformaldehyde. Brains were carefully removed from the skull, post-fixed for 2 h and dissected. Stereological analysis was performed as previously described [57]. Briefly, the left brain hemispheres were cryoprotected in 30 % sucrose, quickly frozen and cut frontally into entire series of 30-μm thick sections on a cryostat (Microm HM550, Germany). Every tenth section was systematically sampled, stained with cresyl violet and used for stereological analysis of the neuron number in the CA1. The hippocampal cell layer CA1 and the striatum (CA1: Bregma −1.22 to −3.80 mm, striatum: Bregma 1.94 to −2.30 mm) were delineated on cresyl violet-stained sections. Using a stereology workstation [Olympus BX51 with a motorized specimen stage for automatic sampling, StereoInvestigator 7 (MicroBrightField, Williston, USA)] and a 100 × oil lens (NA = 1.35), neuronal nuclei were sampled randomly using optical disector probes, and the total number of neurons was subsequently estimated by the fractionator method using a 2-μm top guard zone [66]. The hippocampal cell layer CA1 of heterozygous Tg4-42 mice and wildtype (C57BL/6 J) littermate controls were analyzed at 3, 8 and 12 months of age. In addition, the CA1 and striatum of 8-month-old homozygous Tg4-42 (Tg4-42_hom_) were assessed. All groups were sex- and age-matched (*n* = 3–4 per group).

### RNA extraction, cDNA synthesis and real-time PCR analysis (RT-PCR)

Mice were killed via CO anesthetization followed by cervical dislocation. Mouse brains were rapidly dissected, frozen on dry ice and stored at −80 °C until use. Frozen brain hemispheres were homogenized in 1 ml of Trifast^®^ reagent (Peqlab) per 100 mg tissue using a R50D homogenizer (10 strokes, 800 rpm; CAT). RNA extraction was performed according to the manufacturer’s protocol. RNA was reverse transcribed into cDNA using the First Strand cDNA Synthesis Kit (Fermentas GmbH). Quantitative real-time RT-PCR was performed using a Stratagene MX3000P Real-Time Cycler. For quantification, the DyNamo Flash SYBR Green qPCR Kit containing ROX as an internal reference dye (Finnzymes, Finland) was used. Expression of the transgene was assessed with the following primer set, diluted to a concentration of 10 pmol/μL: 5′-TCCGGCCAGAACGTCGATTC-3′ (forward); 5′-GGAGAAGCAAGA CCTCTGC-3′ (reverse). A mixture of mouse β-actin primers (QuantiTect Primer Assays, Qiagen) served as a control. Statistical analysis of quantitative real-time PCR measurements was done using the Relative Expression Software Tool V2.0.7 (REST 2008) [44].

### Spatial reference memory by Morris water maze

Spatial reference memory in Tg4-42 mice was evaluated using the Morris water maze [39]. Thereby, mice learn to use spatial cues to locate a hidden, circular platform (10 cm) in a circular pool (110 cm diameter) filled with tap water. The water was made opaque by adding non-toxic white paint and maintained at 20 °C for the test duration. The pool was divided into four virtual quadrants that were defined based on their spatial relationship to the platform: left, right, opposite and target quadrants, which contain the goal platform. ANY-Maze video tracking software (Stoelting Co.,Wood Dale, USA) was used to record escape latency, path length, swimming speed and quadrant preference. In order to test whether the groups differed regarding their memory for the former location of the platform in the probe trial, we calculated for each mouse a platform quadrant preference ratio as follows: Time spent in Target Quadrant/(Time spent in Target Quadrant + Time spent in Opposite Quadrant). Preference ratios close to 1 indicate well, whereas ratios close to 0 indicate poor spatial memory.

Heterozygous Tg4-42 mice and wildtype (C57BL/6 J) littermate controls were tested at 3, 8 and 12 months of age. In addition, homozygous Tg4-42 (Tg4-42_hom_) mice were assessed at 3 and 8 months. All groups were sex- and age-matched (*n* = 10–15 mice per group). Each individual mouse was tested at one age only using the cued trials followed by the acquisition training and finalized by the probe trial. After the probe trial, the mice were sacrificed. Testing began with 3 days of cued training. For these trials, the platform was marked with a triangular flag. Mice were introduced into the water at the edge of the pool facing the wall. They were then given 1 min to find the submerged platform. Mice that failed to find the platform in 60 s were gently guided to it. All mice were allowed to sit on the platform for 10 s before being removed from the pool. To prevent hypothermia, all mice were kept in front of a heat lamp for 3 min before being returned to their home cage. Each mouse received four training trials per day with an average inter-trial interval of 15 min. Both the location of the platform and the position at which mice were introduced into the pool changed between trials.

Twenty-four hours after the last day of cued training, mice performed 5 days of acquisition training. For this part of testing, the flag was removed from the platform. In addition to the distal cues existing in the room, proximal visual cues were attached to the outside of the pool. The platform location remained stationary for each mouse throughout training. At the start of every trial, mice were introduced into the pool from one of four predefined entry points. The order in which these entry points were used varied between training days [65]. To avoid quadrant bias, the experimental cohorts were randomly split and trained to find one of two different platform locations. Trials were conducted as during the cued training phase.

Twenty-four hours after the last acquisition trial, a probe test was performed to assess spatial reference memory. The platform was removed from the pool, and mice were introduced into the water from a novel entry point. Mice were then allowed to swim freely for 1 min while their swimming path was recorded.

### Statistical analysis

Differences between groups were tested with unpaired *t* test, one-way analysis of variance (ANOVA) followed by Bonferroni multiple comparison, two-way ANOVA or two-way repeated measures ANOVA followed by Bonferroni multiple comparison or multivariate analysis of variance (MANOVA) as indicated. All data are given as mean ± standard error of the mean (SEM). Significance levels are given as follows: ***p < 0.001; **p < 0.01; *p < 0.05. All statistics were calculated using GraphPad Prism version 5.04 for Windows (GraphPad Software, San Diego, California, USA) and SPSS statistics version 17.0 (IBM, Armonk, New York, USA).

## Results

### Aβ_4-42_ has a high aggregation propensity to form stable aggregates

**Fig. 1:**
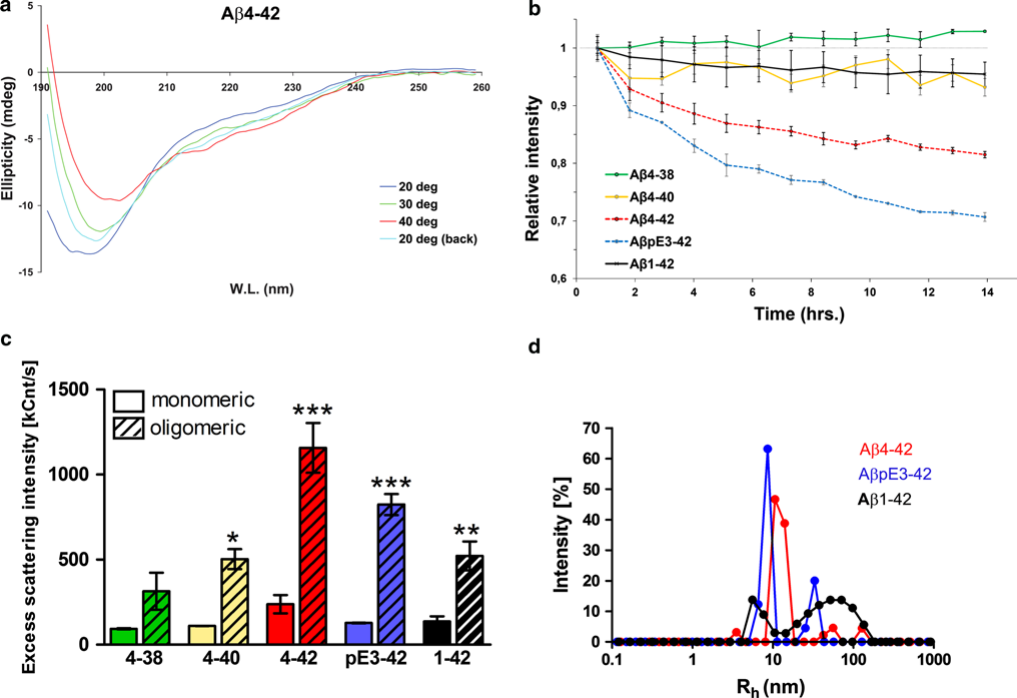
Structural properties of *N*-terminally truncated Aβ. **a** Temperature dependence of far-UV CD spectra of Aβ_4-42_. Measurements were performed at 20, 30 and 40 °C. 20 ° back indicates the measurement after cooling down from 40 to 20 °C. **b** Rates of monomer consumption for different Aβ peptides, probed through a decay in relative intensity of methyl (0.50–1.05 ppm) and aromatic (6.5–8.0 ppm) signals in their 1D ^1^H NMR spectra. **c** Excess light scattering intensity of Aβ variants measured before (*solid bars*) and after (*striped bars*) 24 h of aggregation through dynamic light scattering (DLS). **d** Distribution of hydrodynamic radius (*R*_h_) of Aβ aggregates, derived from their scattering intensity autocorrelation curve in DLS experiments. For Aβ_4-42_ and Aβ_pE3-42_, the main aggregated species had an *R*_h_ of approximately 10 nm

The secondary structure of Aβ_4-42_ was investigated through far-UV CD spectroscopy. At 20 °C, the CD spectrum was characteristic of a disordered state. After temperature increase, Aβ_4-42_ showed reversible changes characterized by the loss of negative intensity at ~198 nm and accompanied with a red shift of the negative peak at ~220 nm. These spectral alterations indicated that Aβ_4-42_ has a high propensity to adopt a folded conformation upon heating (Fig. 1a). The comparison with other Aβ variants is shown in the supplement (Fig. S1–S3). In order to study the aggregation propensities of Aβ variants, we used NMR spectroscopy. For increasing incubation time, ^1^H signals were severely broadened beyond the detection limit of liquid-state NMR. The temporal loss of signal intensity indicates the conversion of NMR-visible monomers and small stable aggregates to large aggregates. As shown in Fig. 1b, the largest decreases in signal intensity were observed for Aβ_pE3-42_ and Aβ_4-42_, which lost ~30 and 20 % of their initial intensities, respectively, after 14 h of incubation at 37 °C. Loss of signal intensity for Aβ_1-42_ and Aβ_4-40_ was around 5 %, while Aβ_4-38_ rose slightly in intensity. These data indicate the following order of aggregation propensity among the five Aβ variants: Aβ_pE3-42_, Aβ_4-42_, Aβ_1-42_/Aβ_4-40_ and Aβ_4-38_. The oligomerization propensities of the five Aβ peptides were then compared through light scattering intensity of the supernatants of the peptide solutions, before and after start of aggregation. Before aggregation, the highest scattering intensity was observed for Aβ_4-42_, followed by Aβ_1-42_, Aβ_pE3-42_, Aβ_4-40_ and Aβ_4-38_ (Fig. 1c). Upon aggregation, all peptides showed a prominent rise in the scattering intensity, reflecting a shift of their size distribution toward larger species. Aβ_4-42_ and Aβ_pE3-42_ had the biggest intensities, followed by Aβ_1-42_ and Aβ_4-40_, then Aβ_4-38_. The scattering intensities of Aβ_4-42_ and Aβ_pE3-42_ were mainly due to small aggregates of ~10 nm in hydrodynamic radius, with smaller but considerable contributions from 30 to 50 nm aggregates (Fig. 1d). On the other hand, Aβ_1-42_ contained small aggregates of 5–10 nm and a wide distribution of aggregates from 20 to 130 nm. Overall, Aβ_4-42_ and Aβ_pE3-42_, and to a lesser extent Aβ_1-42_, have a remarkable tendency to form stable aggregates. The aggregates formed by Aβ_4-42_ and Aβ_pE3-42_ are distinct in size and different from Aβ_1-42_.

### *N*-truncated Aβ variants rapidly aggregate and form fibrils

**Fig. 2:**
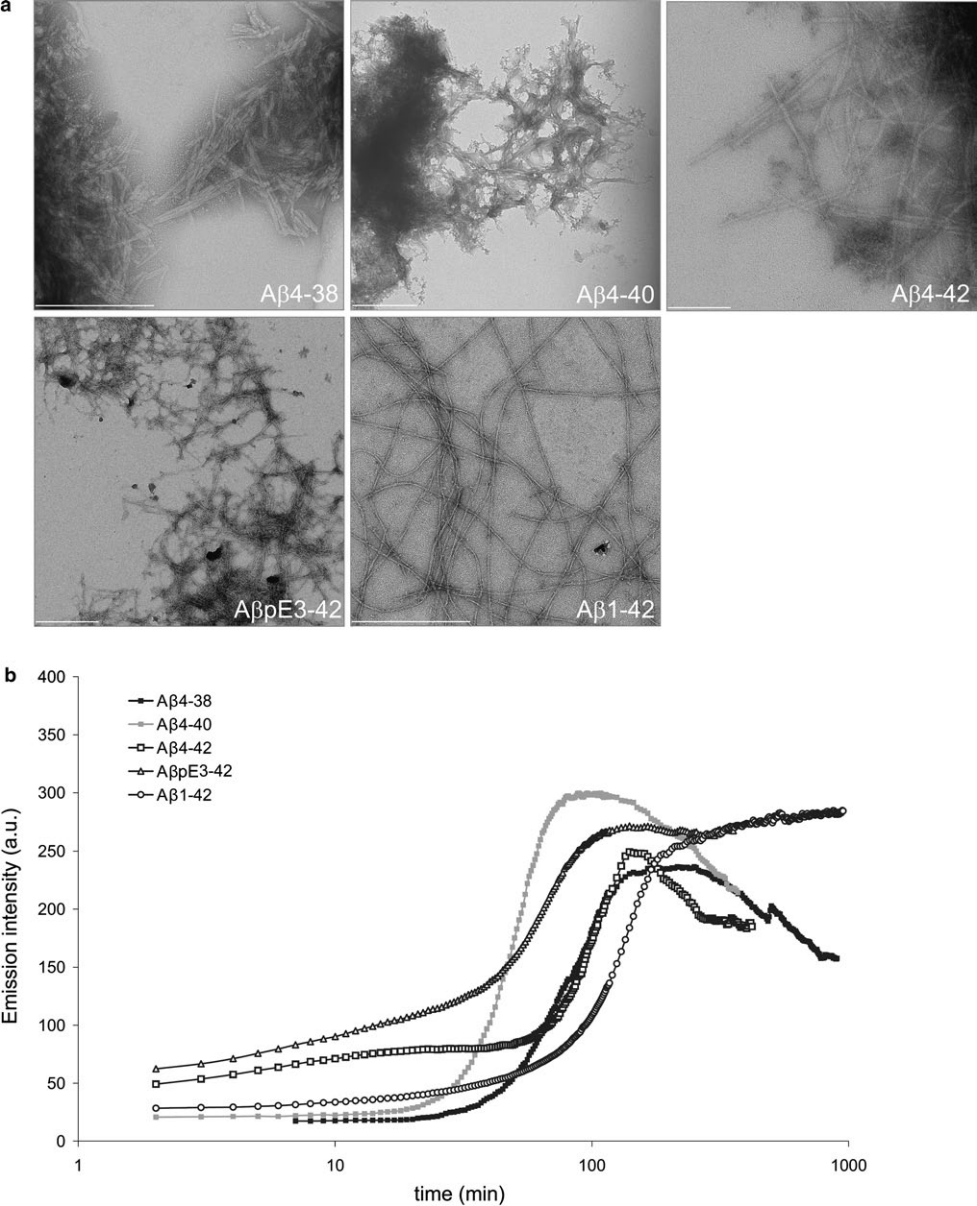
**a** Transmission electron micrograph of Aβ peptides after 3 days of incubation in an aggregation-promoting condition. All Aβ variants formed fibrillar aggregates which tended to clump together in all preparations but Aβ_1-42_. **b** Temporal evolution of Thioflavin T (ThT) fluorescence emission intensity during aggregation of various Aβ peptides. Despite some variation in the kinetics, all five Aβ variants were capable of forming ThT-reactive aggregates. *Scale bars* 200 nm (Aβ_4-42_), 500 nm (all other Aβ variants)

The fibrillar structure of Aβ aggregates, as revealed by transmission electron microscopy (TEM), is displayed in Fig. 2a. Temporal evolution of ThT emission intensity along Aβ aggregation is shown in Fig. 2b. Following lag phases of various durations, sharp rises in ThT emission intensity were observed for all Aβ variants. The maximal ThT intensities reached by the five peptides were similar, although all except Aβ_1-42_ started to decay afterwards. This is probably due to the burial of ThT-binding sites within higher order aggregate states or clumps. This hypothesis is supported by the TEM observations in which the four peptide variants, Aβ_4-38_, Aβ_4-40_, Aβ_4-42_ and Aβ_pE3-42_ often showed clumps of fibrillar aggregates. The observation that all peptides but Aβ_1-42_ formed clumps of fibrils points to the importance of the three *N*-terminal residues for aggregate morphology.

### *N*-truncated Aβ variants elicit stable aggregates

**Fig. 3:**
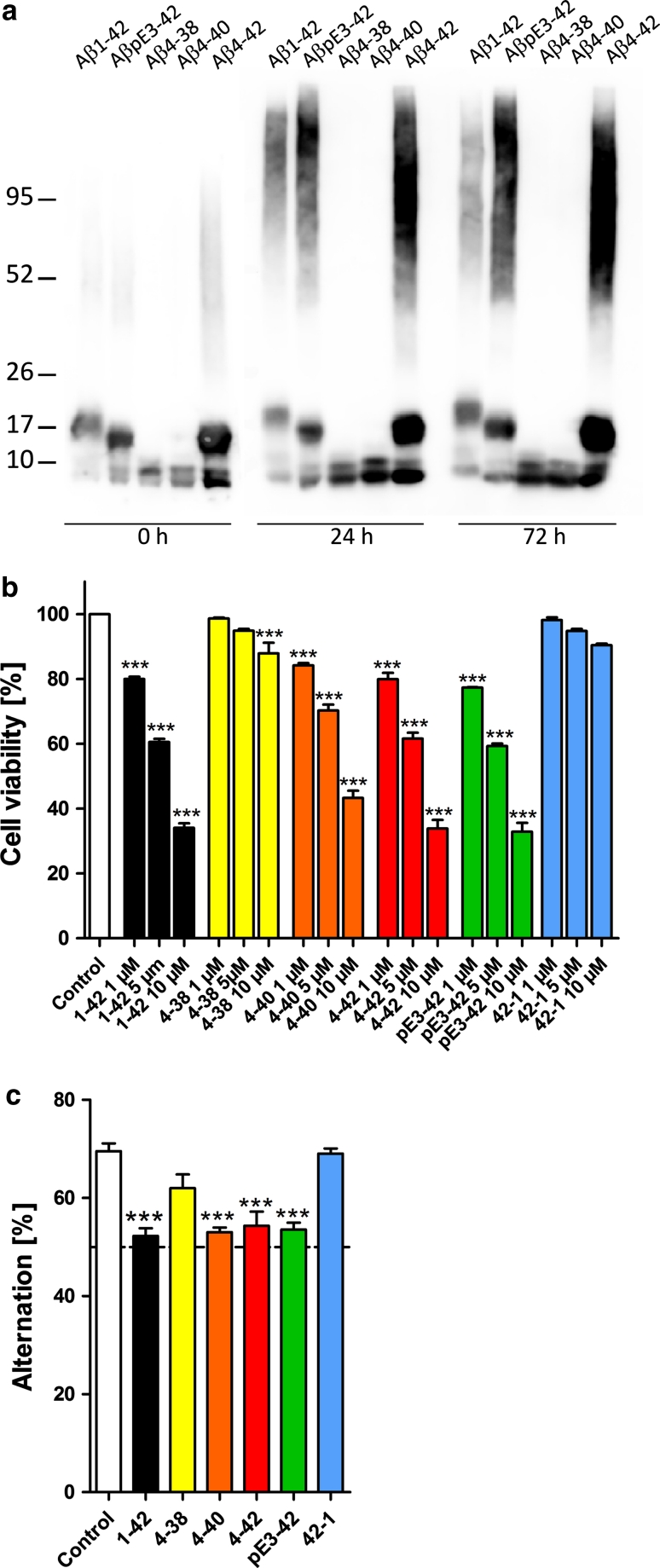
Cellular toxicity of *N*-truncated Aβ peptides. **a** Freshly prepared Aβ_4-38_, Aβ_4-40_,Aβ_4-42_, Aβ_pE3-42_ and Aβ_1-42_ rapidly formed stable aggregates. All peptides displayed dimeric oligomers and monomers under reducing conditions, while Aβ_1-42_, Aβ_pE3-42_ and Aβ_4-42_ also developed SDS-stable tri- or tetrameric oligomers. Aged Aβ_1-42_, Aβ_pE3-42_ and Aβ_4-42_ peptides retained this pattern and exhibited in addition higher molecular weight aggregates. SDS-PAGE Western blot of Aβ peptides using the polyclonal antiserum 24311. **b** *In vitro* toxicity with short-term exposure. Primary neurons were treated with Aβ peptides at different concentrations and analyzed by a cell toxicity assay. Aβ_1-42_ Aβ_pE3-42_ and Aβ_4-42_ demonstrated comparable toxicity profiles followed by Aβ_4-40_. Aβ_4-38_ was only toxic at 10 μM. No toxicity was observed with vehicle control and reverse Aβ_42-1_. **c** *In vivo* toxicity of short-term exposure in wildtype mouse brain. Working memory was assessed after intraventricular injection of Aβ_4-38_, Aβ_4-40_, Aβ_4-42_, Aβ_pE3-42_ and Aβ_1-42_ as well as vehicle and reverse peptide control. Mice injected with Aβ_4-40_, Aβ_4-42_ Aβ_pE3-42_ and Aβ_1-42_ performed at chance level and showed a significant and robust deficit in working memory. Mice treated with Aβ_4-38_ demonstrated normal working memory. The same is true for the reverse peptide Aβ_42-1_ and vehicle control. One-way analysis of variance (ANOVA) followed by Bonferroni multiple comparisons. ***p < 0.001

Freshly dissolved Aβ peptides, for which no specific precautions were taken for monomerization, were subjected to SDS-PAGE (Fig. 3a). Aβ_1-42_, Aβ_pE3-42_, and Aβ_4-42_ displayed monomers and aggregates most likely representing trimer/tetramers, whereas Aβ_4-38_ and Aβ_4-40_ formed monomers, dimers but no other oligomers. After 1 and 3 days of aging, Aβ peptides displayed higher molecular weight oligomers in addition for Aβ_1-42_, Aβ_pE3-42_, and Aβ_4-42_, but not for Aβ_4-38_ and Aβ_4-40_ (Fig. 3a). These Aβ preparations were used to study their acute effects on neuronal function and viability in vitro and in vivo by intraventricular injection into wildtype mouse brain.

### *N*-truncated Aβ variants are toxic in vitro

*In vitro* toxicity was studied in primary neurons using a calcein assay. Treating the cells with freshly prepared Aβ_4-38_, Aβ_4-40_, Aβ_4-42_, Aβ_pE3-42_ and Aβ_1-42_ resulted in a dose-dependent reduction in cell viability (one-way ANOVA, *p* < 0.0001, *F* = 193.6, *df* = 18). After ANOVA, the individual groups were subsequently analyzed using Bonferroni multiple comparisons. The strongest toxic effect was found using Aβ_4-42_, Aβ_pE3-42_ and Aβ_1-42_ followed by Aβ_4-40_ (compared to vehicle control; *p* < 0.0001). Aβ_4-38_ was only toxic at 10 μM. The same was true using a reverse Aβ_42-1_ as an additional control. No significant difference was observed between Aβ_1-42_, Aβ_pE3-42_ and Aβ_4-42_. At 1 μM, there was a difference in the toxicity between Aβ_4-42_ and Aβ_4-40_. At 5 μM, Aβ_1-42_ (*p* < 0.0001) and Aβ_4-42_ (*p* < 0.001) were more potent than Aβ_4-40_. The same was found at 10 μM comparing Aβ_1-42_ (*p* < 0.0001) and Aβ_4-42_ (*p* < 0.0001) with Aβ_4-40_ (Fig. 3b). Phase contrast images of Aβ-treated cells are shown in the supplement (Fig. S4).

### *N*-truncated Aβ variants induce working memory deficits

*In vivo* effects were studied by intraventricular injection of freshly prepared 50 pmol Aβ_4-38_, Aβ_4-40_, Aβ_4-42_, Aβ_pE3-42 and_ Aβ_1-42_ into wildtype mouse brain. Working memory was assessed using Y-maze (Fig. 3c). There was a significant treatment effect (one-way ANOVA, *p* < 0.001, *F* = 19.98, *df* = 6). After ANOVA, the individual groups were then analyzed using Bonferroni multiple comparisons. The alternation rate was significantly reduced after injection of Aβ_4-40_ (*p* < 0.01) and Aβ_4-42_ (*p* < 0.01) reaching chance level. Mice injected with Aβ_4-38_ behaved like vehicle controls and learned well.

### Aβ_4-42_ expression in Tg4-42 mice

**Fig. 4:**
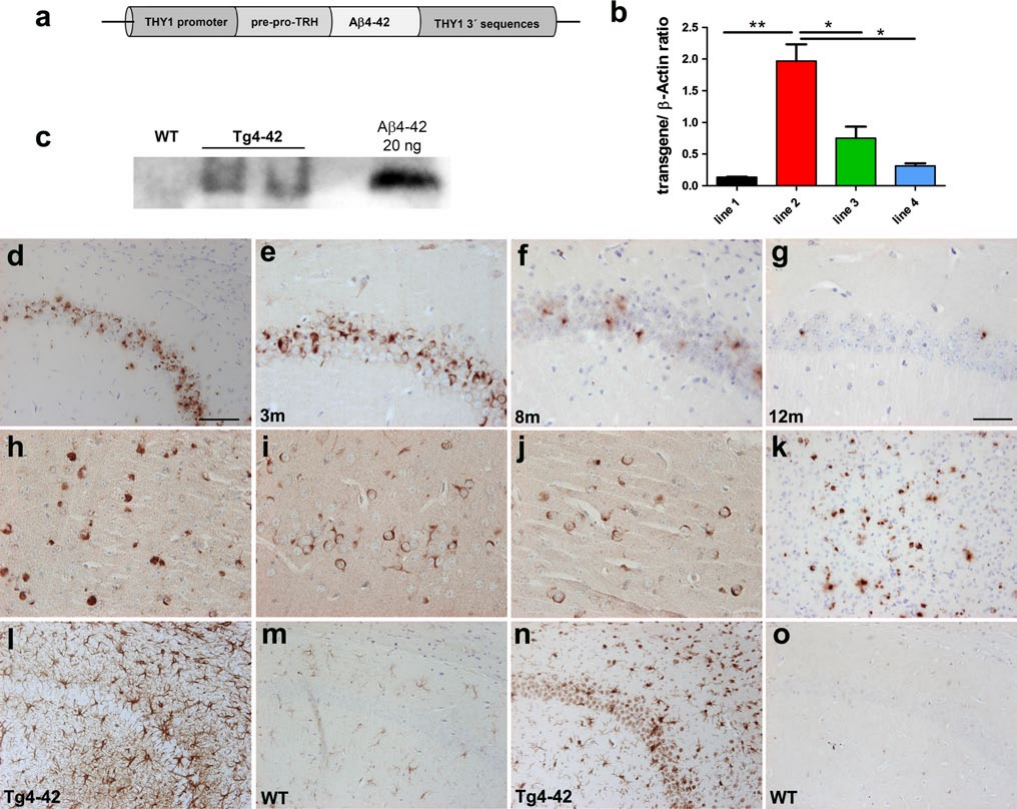
Analysis of transgenic mice expressing Aβ_4-42_. **a** Scheme of transgene expression vector with THY1 promoter, signal peptide of pre-pro-thyrotropin releasing hormone (TRH), Aβ_4-42_ and THY1 3′ sequences. **b** Quantitative RT-PCR analysis of four different transgenic mouse lines producing Aβ_4-42_ showing variable transgene expression levels. Line 2 exhibited significantly the highest transgene expression (*p* < 0.01 unpaired *t* test compared to the other lines). This line was chosen for further breeding and renamed Tg4-42. **c** Western blot analysis of whole brain TBS lysates (40 μg total protein loaded) using pan-Aβ antibody 4G8 of 5-month-old hemizygous Tg4-42 mice and a wildtype control. **d, o**–**n** Immunohistochemical staining profile of Tg4-42 mice expressing Aβ_4-42_. **d** Abundant intraneuronal Aβ immunoreactivity was found in the CA1 pyramidal cell layer of the hippocampus in 3-month-old hemizygous Tg4-42 mice (polyclonal antiserum 24311). Aβ42 immunostaining in CA1 at 3 **e**, 8 **f** and 12 **g** months of age in hemizygous Tg4-42 mice with an age-dependent reduction in positive cells. Other brain regions with Aβ42 staining were occipital cortex (**h**), piriform cortex **i**, striatum **j** and superior colliculus **k**. In addition, increased astrogliosis with GFAP staining **l** and microgliosis with IBA1 staining **n** was observed as early as 2 months of age in hemizygous Tg4-42 mice. No significant astro and microgliosis were seen in wildtype (WT) controls (GFAP **m**, IBA1 **o**). *Scale bar* in **d** 100 μm (**d**, **k**–**o**); *scale bar* in **g**: 50 μm (**e**–**j**)

In order to analyze the effect of chronic exposure of Aβ_4-42_ in mouse brain, the Tg4-42 transgenic mouse line was developed. The transgene drives the neuronal expression of human Aβ_4-42_ fused to the murine thyrotropin releasing hormone (TRH) signal peptide under the control of the Thy-1 promoter (Fig. 4a). The fusion peptide routes Aβ_4-42_ through the secretory pathway enabling its extracellular release [68]. After pronuclear injection, eight transgenic founder lines were obtained and crossed with C57BL/6 J wildtype mice. Offspring from four of the successfully breeding founders were further examined by RT-PCR in order to identify the line with the highest expression level. Line 2 showed the highest expression level of the transgene relative to the other three lines (Fig. 4b; unpaired *t* tests, *p* < 0.05 line 2 versus line 3 and 4; *p* < 0.01 line 2 versus line 1). No significant differences in transgene levels were identified between lines 1, 3 and 4. As a result, mouse line 2 was selected for further characterization and renamed Tg4-42. Protein expression was assessed using Western blot of Tg4-42 mouse brain (Fig. 4c).

**Fig. 5:**
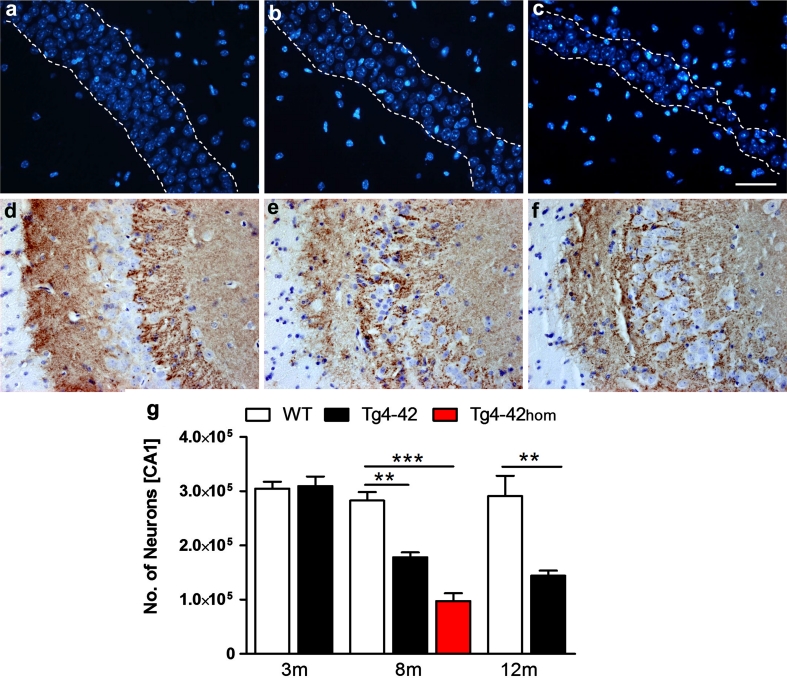
Age- and dose-dependent neuron loss in hippocampus in Tg4-42 mice. DAPI staining (**a**–**c**) revealed a loss in neuron number in the CA1 layer of the hippocampus at the age of 8 months between hemizygous **b** Tg4-42 and **a** age-matched WT mice. **c** A more pronounced neuron loss was apparent in homozygous Tg4-42 mice. **d**–**f** Synaptophysin staining showed an altered synaptic patterning in the CA3 region of the hippocampus in hemizygous and more pronounced in homozygous Tg4-42 mice at the age of 8 months. **g** Quantification using unbiased stereology. *Scale bars* **a**–**c** 100 μm; **d**–**f** 50 μm; one-way analysis of variance (ANOVA) followed by Bonferroni multiple comparisons. *p < 0.05, **p < 0.01

Tg4-42 brain sections showed strong immunoreactivity predominantly in the CA1 region of the hippocampus beginning at the age of 2 months (Fig. 4d). CA1 Aβ expression declined during aging (Fig. 4d–g) due to neuron loss in CA1 (Fig. 5d). No difference was detected in Aβ expression between the polyclonal antiserum 24311 recognizing pan-Aβ and the Aβ42-specific antiserum (Fig. 4d, e). Other brain regions with Aβ42 staining were occipital cortex (Fig. 4h), piriform cortex (Fig. 4i), striatum (Fig. 4j) and superior colliculus (Fig. 4k). Reactive microglia and astroglia demonstrated hippocampal neurodegeneration beginning at 2 months of age with no gliosis in wildtype littermate control mice (Fig. 4l–o).

### Long-term exposure to *N*-truncated Aβ_4-42_ induces neuron loss

Obvious age- and dose-dependent neuron loss was seen in the hippocampus CA1 region of aged hemizygous and homozygous Tg4-42 mice (Fig. 5a–c, g) (one-way ANOVA, *p* = 0.001, *F* = 39.07; *df* = 2). Hemizygous Tg4-42 showed a 38 % neuron loss (mean = 178,000, SEM ± 9,083; *p* = 0.01) that was even more pronounced in homozygous Tg4-42 mice with a 66 % decline (mean = 97,600, SEM ± 14,300, *p* = 0.001) compared to WT controls (mean = 282,700, SEM ± 15,670) at 8 months of age (Fig. 5g). Hemizygous 12-month old Tg4-42 mice (mean = 144,100, SEM ± 9,540) displayed a 49 % neuron loss compared to same age WT (mean = 291,100, SEM ± 37,530) controls (Fig. 5g). Between 8 and 12 months, the neuron loss in hemizygous mice increased by 23 %. At 3 months of age, no difference in the number of neurons was observed between hemizygous Tg4-42 (mean = 309,600, SEM ± 17,570) mice and wildtype littermates (mean = 304,800, SEM ± 12,560). The striatum was unaffected and did not show any significant neuron loss in 8-month-old Tg4-42_hom_ mice (Fig. S5) supporting a primary role of the hippocampus for the spatial reference memory deficits in the Tg4-42 mouse model. Synaptophysin staining showed an altered synaptic patterning in the CA3 region of the hippocampus reflecting a possible network disturbance of neurons that are directly linked to the CA1 area. This synaptic alteration was seemingly more pronounced in homozygous as compared to hemizygous Tg4-42 mice at the age of 8 months (Fig. 5d–f).

### Long-term exposure to *N*-truncated Aβ_4-42_ induces spatial memory deficits

Spatial reference memory was assessed in wildtype (WT), hemizygous and homozygous Tg4-42 mice, expressing Aβ_4-42_, using the Morris water maze. Hemizygous Tg4-42 mice were tested at 3, 8 and 12 months of age in comparison to WT mice. In addition, homozygous Tg4-42 (Tg4-42_hom_) mice were tested at 3 and 8 months of age. First, mice performed cued training with a marked platform to familiarize with the pool and to rule-out effects from possible motor or sensory deficits. WT, Tg4-42 and Tg4-42_hom_ mice showed progressively decreased escape latencies at all ages tested (data not shown). The cued training revealed that all mice had intact vision and the appropriate motor abilities to swim.

**Fig. 6:**
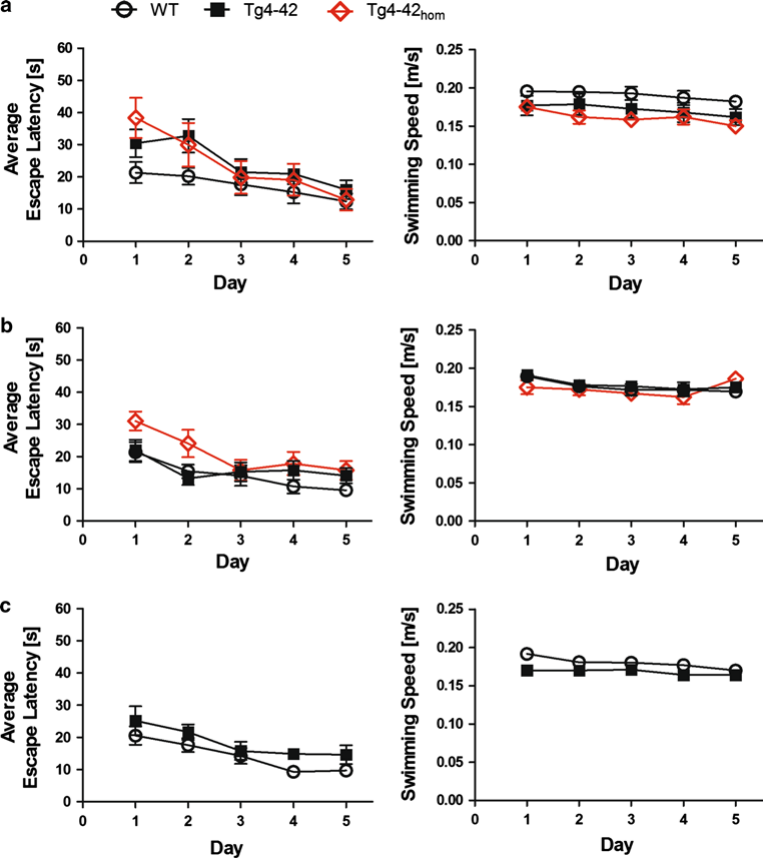
Spatial learning was assessed using acquisition training of the Morris water maze. Heterozygous Tg4-42 mice and wildtype (C57BL/6 J) littermate controls were tested at **a** 3, **b** 8 and **c** 12 months of age. In addition, homozygous Tg4-42 mice (Tg4-42_hom_) were assessed at 3 and 8 months. Each group was sex- and age-matched and contained 10–15 animals. Animals tested underwent acquisition training to learn to use distal and proximal cues to navigate a direct path to a hidden platform. **a**–**c** Escape latencies decreased progressively over 5 days of training for wildtype, Tg4-42 and Tg4-42_hom_. Swimming speed was not affected in all mice tested. *m* age in months

Twenty-four hours after the cued training, mice were subjected to acquisition training in order to test their learning abilities to find the location of a submerged platform by using distal and proximal cues. Across the 5 days of acquisition the animals, irrespective of genotype and age, showed a significant decrease in the escape latencies (Fig. 6a–c) and the path lengths (data not shown) to reach the hidden platform (two-way repeated measures ANOVA, main effect of *days*: Escape latency: *F* (4,320) = 26.920; *p* < 0.001; Path length: *F* (4,320) = 28.148; *p* < 0.001). In contrast, no changes in the swimming speed of the animals were evident over the 5 days (Fig. 6d–f, two-way repeated measures ANOVA, *F* (4,320) = 0.596; *p* < 0.666). There was a significant main effect of the factor *age* on both escape latencies (two-way repeated measures ANOVA, *F* (2,80) = 3.982; *p* = 0.022) and the path lengths (two-way repeated measures ANOVA *F*(2,80) = 4.278; *p* = 0.017), whereas swimming speed was not significantly affected by the age of the animals (two-way repeated measures ANOVA *F* (2,80) = 2.550; *p* = 0.084).

These results suggest that younger animals performed superior in comparison to older animals. There were no age-related motor deficits as the swimming velocity was not different at all ages.

We also found a significant main effect of *genotype* for escape latencies (two-way repeated measures ANOVA, *F* (2,80) = 4.905; *p* = 0.010) and a trend for a significant difference for path lengths (two-way repeated measures ANOVA *F*(2,80) = 3.027; *p* = 0.054). In contrast, no significant main effect of *genotype* was found for the swimming speed readout (two-way repeated measures ANOVA, *F* (2,80) = 1.238; *p* = 0.296). These results suggest that Aβ_4-42_ expression in the mouse impairs spatial learning in the Morris water maze. No significant *genotype x age* interaction was found (two-way repeated measures ANOVA, Escape latency: *F* (2,80) = 0.718; *p* = 0.491; Path length: *F* (2,80) = 0.297; *p* = 0.744).

**Fig. 7:**
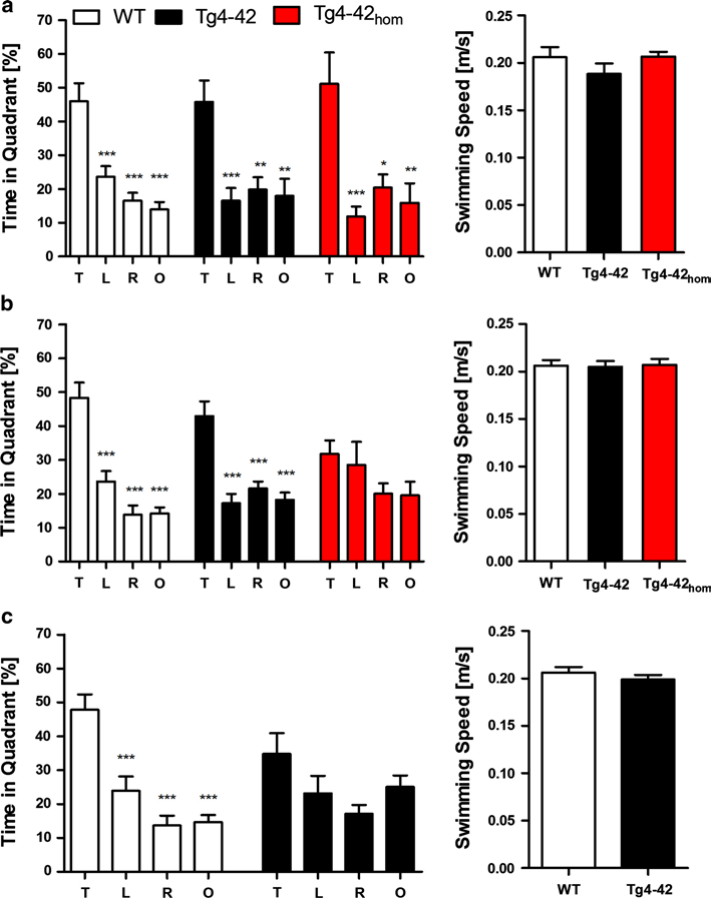
Memory deficits in aged Tg4-42 mice shown in the probe trial of the Morris water maze. Hemizygous Tg4-42 mice and WT (C57BL/6 J) littermate controls were tested at **a** 3, **b** 8 and **c** 12 months of age. In addition, homozygous Tg4-42 (Tg4-42_hom_) mice were assessed at 3 and 8 months. Each group was sex- and age-matched and contained 10–15 mice. The probe trial was given at the end of the learning phase (acquisition training) to assess spatial reference memory. Quadrant preference and swimming speed for the first 30 s of the probe trial were analyzed. **a** Tg4-42, Tg4-42_hom_ and WT mice showed no impairment in spatial reference memory at 3 months of age. Both groups spent a significant greater percentage of the time in the target quadrant. **b** The probe trial revealed significantly reduced learning behavior for Tg4-42_hom_ mice at 8 months of age as they showed no preference for the target quadrant. In contrast, hemizygous Tg4-42 and WT mice had no learning deficits at this age. **c** At 12 months of age, hemizygous Tg4-42 mice showed no quadrant preference revealing an impaired spatial reference memory. However, WT mice still learned, as they had a significant preference for the target quadrant. **a**–**c** No differences in swimming speed between WT, Tg4-42 and Tg4-42_hom_ were detected at any tested age. *T* target quadrant, *L* left quadrant, *R* right quadrant, *O* opposite quadrant, *m* age in months. Quadrant preference: Two-way analysis of variance (ANOVA) followed by Bonferroni multiple comparisons. Swimming speed: unpaired *t*-test. ***p < 0.001; **p < 0.01

Twenty-four hours after the last acquisition trial, a probe trial was performed to assess spatial reference memory. At 3 months of age, WT,Tg4-42 and Tg4-42_hom_ mice displayed a significant preference for the target quadrant, as indicated by the percentage time spent in different quadrants of the pool (Fig. 7a, two-way ANOVA, *p* < 0.0001, *F* = 29.12, *df* = 3 for quadrants; WT: *p* < 0.001 target versus left quadrant, *p* < 0.0001 target versus right and opposite quadrant; Tg4-42: *p* < 0.0001 target versus left, *p* < 0.001 target versus right and opposite quadrant; Tg4-42_hom_: *p* < 0.001 target versus left and opposite quadrant, *p* < 0.01 target versus right quadrant; two-way ANOVA, *p* = 0.5942, *F* = 0.7710, *df* = 6 for interaction between quadrants and genotype). No quadrant preference was found for the 8-month-old homozygous Tg4-42 mice, while WT and Tg4-42 mice still demonstrated significant preference for the target quadrant at that time point (Fig. 7b, two-way ANOVA, *p* < 0.0001, *F* = 28.80, *df* = 3 for quadrants; WT: *p* < 0.0001 target versus all other quadrants; Tg4-42: *p* < 0.0001 target versus all other quadrants; Tg4-42hom: *p* > 0.05 target versus all other quadrants; two-way ANOVA, *p* = 0.0049, *F* = 3.267, *df* = 6 for interaction between quadrants and genotype). Twelve-month-old WT mice spent a significant higher percentage of time in the target quadrant in comparison to the other quadrants. However, 12-month-old Tg4-42 mice displayed no significant preference for the target quadrant indicating a robust deficit in spatial reference memory (Fig. 7c, two-way ANOVA, *p* < 0.0001, *F* = 15.73, *df* = 3 for quadrants WT: *p* < 0.001 target versus left quadrant, *p* < 0.0001 target versus right and opposite quadrant; Tg4-42: *p* > 0.05 target versus all other quadrants; two-way ANOVA, *p* = 0.0371, *F* = 2.955, *df* = 3 for interaction between quadrants and genotype). Swimming speed did not differ during the probe trial between the groups or the different ages tested (Fig. 7a–c). A multivariate ANOVA performed on target versus opposite quadrant preference ratios yielded a main effect of the factor *genotype* (MANOVA, *F* (2,75) = 5.682; *p* = 0.005) but no main effect of the factor *age* (MANOVA, *F* (2,75) = 1.367; *p* = 0.261). However, there was a significant *genotype x age* interaction for the readout preference ratio (MANOVA, *F* (2,75) = 4.204; *p* = 0.019). These results suggest that the detrimental effects of Aβ_4-42_ expression on spatial learning in the mouse aggravates in the course of aging.

In sum, the results of the acquisition phase and the probe trial suggest that Aβ_4-42_ expression in the mouse impairs spatial learning in the Morris water maze as reflected by the absence of a preference for the target quadrant as compared to the remaining quadrants during the probe trial. This spatial learning deficit is much more pronounced in old as compared to young Tg4-42 mice.

## Discussion

In vitro and in vivo analysis of amyloid deposits in AD revealed N- and C-terminal variants of the Aβ peptide [33, 35, 48]. Masters et al. [33] discovered that the majority (64 %) of the peptides in amyloid plaques of AD begin with a phenylalanine residue corresponding to position 4 of the full-length sequence. Moreover, they detected dimeric and tetrameric (termed A8 and A16, respectively) Aβ aggregates from the HPLC separations of plaques from AD having the same ragged NH_2_-terminal ends. The importance of Aβ_4-42_ was later supported by showing that Aβ_4-42_ represents a dominant fraction in the hippocampus and cortex of AD patients using immunoprecipitation and mass spectrometry [47]. In addition, Lewis et al. [31] reported that Aβ_4-42_ is a relatively abundant species in AD, aged controls and vascular dementia patients. Other groups identified Aβ_11-42_ as the only *N*-truncated species [41]. Mori and colleagues described the presence of Aβ peptides (15–20 % of the total Aβ) bearing a pyroglutamate residue at the *N*-terminus. By using pyroglutamate amino peptidase, they were able to unravel the amino acid terminal, which is blocked by the lactam ring and thus resistant to any other peptidase for Edman sequencing used in previous reports [37]. Since then, the interest in dissecting the temporal and spatial deposition of pyroglutamate Aβ increased. Saido et al. [54] showed by immunohistochemical and biochemical means that Aβ_pE3_ is present in equivalent or larger amounts than full-length Aβ in senile plaques. This was further confirmed by another study on water-soluble Aβ demonstrating the presence of Aβ_pE3-42_ in AD and Down syndrome (DS) as a dominant fraction [52]. In line with the previous findings, testing extracts from AD and DS frontal cortex using ELISA revealed that levels of Aβ_pE3_ and isomerized Aβ species ending at amino acid 42 were higher than those ending with amino acid 40 [15, 18]. This was further confirmed by the finding that Aβ_pE3-42_ constituted 25 % of the total Aβ_x-42_ in plaques of AD brains [15]. It was reported that unmodified Aβ_1-40_ and Aβ_1-42_ can be modified into Aβ_pE3_ after being injected into rat brain indicating that rat brains harbor the enzymes required for *N*-terminal truncation and pyroglutamate formation [63]. Analysis of water soluble Aβ in AD, DS as well as non-demented elderly brain specimens indicated the presence of Aβ_1-42_, Aβ_pE3-42_ and Aβ_pE11-42_. Russo et al. [53] showed that cases with a PS1 mutations develop a higher ratio of water-soluble Aβ_pE3-42_ and Aβ_pE11-42_ to full-length Aβ_1-42_ in comparison to sporadic AD cases.

In addition, biochemical studies showed that Aβ peptides isolated from AD brains were post-translationally modified by isomerization and racemization [26, 38]. Isomerized Aβ at the seventh amino acid was suggested to comprise a major fraction of Aβ in neuritic plaques [50]. Both modifications have been shown to accelerate peptide aggregation and fibril formation [38, 62, 64]. Other modifications include metal-induced oxidation [11] or phosphorylation [24, 25, 36].

*N*-terminal deletions enhance Aβ aggregation and toxicity in relation to full-length Aβ [45]. Pike et al. [45] compared Aβ peptides with initial residues at positions 1, 4, 8, 12, and 17 and ending with residue 40 or 42 using circular dichroism spectra. They reported a predominant β-sheet conformation, fibrillar morphology under transmission electron microscopy, and significant toxicity in cultures of rat hippocampal neurons. Our data extend these observations and show that soluble aggregates have specific features responsible for their neurotoxicity. We demonstrated that all five Aβ variants studied (Aβ_4-38_, Aβ_4-40_ Aβ_4-42_, Aβ_1-42_ and Aβ_pE3-42_) are unstructured in the monomeric state. However, upon heating the Aβ variants showed a high propensity to form folded structures, in particular the three most toxic variants Aβ_pE3-42_, Aβ_1-42_ and Aβ_4-42_. In addition, monomeric Aβ_4-42_ and Aβ_pE3-42_ were rapidly converted to soluble aggregated species. The soluble aggregates are capable of converting to ThT-reactive fibrillar aggregates with Aβ_4-42_ and Aβ_pE3-42_ showing significant ThT-reactivity already during the nucleation phase of aggregation. The observation that the propensity of Aβ_4-42_ to form aggregates is more pronounced than the *N*-terminally intact Aβ_1-42_ peptide suggests that Aβ_4-42_ aggregation may precede Aβ_1-42_ aggregation in the in vivo condition.

Small, soluble Aβ_1-42_ oligomers ranging in size from dimers to dodecamers have been found as key drivers of neurotoxicity in vitro and in vivo [6, 28, 30, 43, 61, 71]. Increased *C*-terminal length of Aβ (from Aβ_1-40_ to Aβ_1-42_) enhances aggregation, early deposition and promotes the toxicity of Aβ [2, 19, 45] suggesting that Aβ_1-42_ aggregates represent the major toxic factor [3]. At the same time, there is increasing evidence that *N*-truncated species, such as Aβ_pE3-42_, may contribute to AD-typical behavioral deficits [20, 56]. The increased hydrophobicity of the *N*-terminal part upon removal of the first three residues may influence the interaction of Aβ aggregates with cellular membranes and modulate its cytotoxic properties. Here we show evidence that short-term exposure to aggregated Aβ_4-x_ peptides triggers neuron loss in primary cortical cultures, with the strongest effect for Aβ_4-42_ followed by Aβ_4-40_ and Aβ_4-38_. Intracerebral infusion of Aβ_1-42_ and Aβ_pE3-42_ oligomers has been repeatedly shown to affect hippocampus-dependent behavior assessed by working memory behavioral testing [6, 71]. Our studies are well in line with these observations. We demonstrate that Aβ_4-40_ and Aβ_4-42_, but not Aβ_4-38_, have comparable detrimental effects using the same concentration as previously employed for Aβ_1-42_ and Aβ_pE3-42_ oligomers [71].

Levels of *N*-truncated and modified Aβ are known to vary between AD mouse models. In Tg2576 mice, truncated and modified Aβ isoforms do not appear before 1 year of age and comprise approximately 5 % of total Aβ [22]. Aβ_pE3_ and other modified forms of Aβ were reported to be absent in APP23 mice until almost 2 years of age [27] or low in PS2APP mice [13]. Using another approach, Maeda and colleagues demonstrated that the localization and abundance of [^11^C]PIB autoradiographic signals were closely associated with Aβ_pE3_ plaques in AD and different APP transgenic mouse brains. This observation suggests that the [^11^C]PIB-PET retention signal depends on the accumulation of specific Aβ subtypes [32]. Interestingly, significant brain-area-specific neuron loss develops in both APP/PS1KI and 5XFAD mice [4, 7–9, 21, 42]. The TBA42 mouse model, like the TBA2, TBA2.1 and TBA2.2 models [1, 68], expresses Aβ_3Q-42_ starting with an *N*-terminal glutamine (Q) residue at position three of Aβ. Glutamine was used instead of the naturally occurring glutamate since it is a better substrate for both the spontaneous and enzymatically catalyzed conversion of Aβ_3-42-_ into Aβ_pE3-42_ [10]. The degree of conversion was not determined in the TBA2, TBA2.1 and TBA2.2 mice. Therefore, unmodified *N*-truncated Aβ_3-42_ could also contribute to the observed pathology and behavioral phenotype. Using mass spectrometric analysis, we could previously demonstrate that 5XFAD mice already exhibit high amounts of Aβ_pE3-42_ and other Aβ isoforms. Besides Aβ_1-42_, the following peptides were also identified in 5XFAD mice, in order of abundance: Aβ_1-40_, Aβ_4-42_, Aβ_5-42_, Aβ_pE3-42_ and Aβ_3-42_. The appearance of an exceedingly heterogeneous population of *N*-truncated and modified Aβ peptides in 5XFAD mice is in line with previous observations made in the APP/PS1KI mouse model [7].

In order to investigate the long-lasting neurotoxic effect of Aβ_4-42_, we generated transgenic mice expressing Aβ_4-42_ (Tg4-42 mouse line). Tg4-42 mice develop severe hippocampus neuron loss and spatial reference memory deficits. These data are corroborated by previous mouse models expressing full-length mutant APP. For example, APP/PS1KI mice exhibit neuron loss in the CA1 region of the hippocampus [4, 7], the frontal cortex [8], and in distinct cholinergic nuclei [9]. This model is characterized by age-dependent accumulation of heterogeneous *N*-terminal truncated Aβ peptides with Aβ_4-42_ being one of the most abundant variants. In 5XFAD, another mouse model expressing mutant APP and PS1 [42], a heterogeneous mixture of full-length, *N*-truncated and modified Aβ peptides, including Aβ_4-42_, were found [70]. The pathological events observed in the APP/PS1KI and 5XFAD mouse models might be at least partly triggered by *N*-terminal truncated Aβ_x-42_. Neuron loss and an associated severe neurological phenotype were found in a transgenic mouse model expressing only *N*-truncated Aβ_pE3-42_ [69], supporting the concept that *N*-truncated Aβ is neurotoxic. At present it is unclear whether *N*-truncated Aβ starting at position four is derived from the full-length Aβ_1-42_ or directly from the amyloid precursor protein. However, once it is generated it might actively participate in the amyloid cascade.

The Tg4-42 model represents the first mouse model expressing exclusively *N*-truncated Aβ_4-42_. At 8 months homozygous Tg4-42 mice showed severe neuron loss in the CA1 region (66 %) accompanied by impaired spatial memory. In spite of a 38 % neuron loss in the CA1 at 8 months of age hemizygous Tg4-42 mice demonstrated no deficits in learning. Broadbent et al. [5] examined the relationship between hippocampal lesion size and spatial memory in rats. Spatial memory impairment started after bilateral dorsal hippocampal lesions that encompassed 30–50 % total volume, and as lesion size increased from 50 to 100 % of total hippocampal volume, performance was similarly impaired. In addition, Moser et al. [40] claimed that only 20–40 % of the total hippocampus is required for efficient spatial learning. These findings show that the hippocampus is important for spatial memory albeit a significant neuron loss can be compensated. Our findings are in good agreement with these observations as a 38 % neuron loss in the CA1 of the hippocampus in 8-month-old hemizygous Tg4-42 mice has no consequence on spatial reference memory performance. However, homozygous Tg4-42 mice with a 66 % neuron loss demonstrate significant impaired spatial learning in the Morris water maze. Both the age-dependent deficits in spatial reference memory and the severe hippocampal neuron loss in homozygous Tg4-42 mice are compatible with AD-typical changes.

The mode of Aβ and in particular Aβ_4-42_ toxicity is currently not clear. It has been suggested that membrane permeabilization by amyloid oligomers may initiate a common group of downstream pathologic processes, including intracellular calcium dyshomeostasis, production of reactive oxygen species, altered signaling pathways, and mitochondrial dysfunction that represent key effectors of cellular dysfunction and cell death [12]. Naturally secreted Aβ oligomers may directly impair synaptic function and have been shown to block hippocampal long-term potentiation (reviewed in [14]). Recently, it was demonstrated that Aβ_1-42_ oligomers trigger cell surface receptor clustering near or within synapses, leading to mGluR5 dysfunction [49]. Our finding that soluble and aggregated Aβ_4-42_ species are as toxic as Aβ_1-42_ suggests that similar mechanisms might be active in the case of Aβ_4-42_ aggregates.

The controversy among different studies regarding the predominant species and their contribution to the pathology of AD might reflect differences in the brain regions analyzed, imbalances in age and disease stages of the recruited cases, different protocols utilized and the characteristics of the peptides under investigation. Our current and previously published data demonstrate that both Aβ peptides Aβ_4-42_ and Aβ_pE3-42_ are likely playing a dominant role in triggering AD pathology. Which one of these two peptides might be accumulating first in AD is presently unclear as no antibody recognizing the *N*-terminus of Aβ_4-42_ is available. In the present study, we revealed actions of human Aβ_4-42_ in the mouse brain that are consistent with a role for Aβ_4-42_ in the pathogenesis of AD in humans. Soluble Aβ_4-42_ aggregates triggered neuron death in primary cortical neurons and significantly affected the working memory phenotype in wildtype mice after intraventricular injection. Aβ_4-42_ aggregates showed a high aggregation propensity and stability. Finally, long-term exposure to Aβ_4-42_ induced neuron loss and behavioral deficits in transgenic Tg4-42 mice.
